# Supplementary material for: The Burden of Hidradenitis Suppurativa Signs and Symptoms in Quality of Life: Systematic Review and Meta-Analysis
Source: Int J Environ Res Public Health. 2021 Jun 22;18(13):6709. doi: 10.3390/ijerph18136709 (PMC8293810; doi:10.3390/ijerph18136709)
Supplement: Supplementary file 1 [file ijerph-18-06709-s001.zip › ijerph-1248957-supplementary.pdf]

Table S1. Quality assessment of the included studies.

Study, site and year	1. Was the research question or objective in this paper clearly stated?	2. Was the study population clearly specified and defined?	3. Was the participation rate of eligible persons at least 50%?	4. Were all the subjects selected or recruited from the same or similar populations (including the same time period)? Were inclusion and exclusion criteria for being in the study prespecified and applied uniformly to all participants?	5. Was a sample size justification, power description, or variance and effect estimates provided?	6. For the analyses in this paper, were the exposure(s) of interest measured prior to the outcome(s) being measured?	7. Was the timeframe sufficient so that one could reasonably expect to see an association between exposure and outcome if it existed?	8. For exposures that can vary in amount or level, did the study examine different levels of the exposure as related to the outcome (e.g., categories of exposure, or exposure measured as continuous variable)?	9. Were the exposure measures (independent variables) clearly defined, valid, reliable, and implemented consistently across all study participants?	10. Was the exposure(s) assessed more than once over time?	11. Were the outcome measures (dependent variables) clearly defined, valid, reliable, and implemented consistently across all study participants?	12. Were the outcome assessors blinded to the exposure status of participants?	13. Was loss to follow-up after baseline 20% or less?	14. Were key potential confounding variables measured and adjusted statistically for their impact on the relationship between exposure(s) and outcome(s)?	Quality Rating
**Alavi et al. Canada 2017 [37]**	Yes	Yes	NR	Yes	No	NA	NA	NA	Yes	NA	Yes	NA	NA	Yes	Fair
**Cuenca-Barrales et al. Spain.2019 [25]**	Yes	Yes	Yes	Yes	No	NA	NA	NA	Yes	NA	Yes	NA	NA	Yes	Fair
**Cuenca-Barrales and Molina-Leyva. Spain.2020 [24]**	Yes	Yes	Yes	Yes	No	NA	NA	NA	Yes	NA	Yes	NA	NA	Yes	Fair
**Frings et al. Germany. 2019 [26]**	Yes	Yes	NR	Yes	No	NA	NA	NA	Yes	NA	Yes	NA	NA	No	Fair

**Huilaja et al. Finland. 2020 [27]**	Yes	No	NR	NR	No	NA	NA	NA	No	NA	Yes	NA	NA	No	Poor
**Jørgensenet al. Germany. 2020 [28]**	Yes	Yes	NR	Yes	No	NA	NA	NA	Yes	NA	Yes	NA	NA	Yes	Fair
**Kaaz et al. Poland. 2018 [29]**	Yes	Yes	NR	Yes	No	NA	NA	NA	Yes	NA	Yes	NA	NA	Yes	Fair
**Kirby et al. Denmark and USA. 2021 [30]**	N0	Yes	NR	Yes	No	NA	NA	NA	Yes	NA	No	NA	NA	No	Fair
**Krajewski PK et al. Poland. 2021 [31]**	Yes	Yes	NR	Yes	No	NA	NA	NA	Yes	NA	Yes	NA	NA	Yes	Fair
**Machado et al. Canada. 2021 [38]**	Yes	No	NR	NR	No	NA	NA	NA	No	NA	No	NA	NA	No	Fair
**Matusiak et al. Poland 2018. [32]**	Yes	Yes	NR	Yes	No	NA	NA	NA	Yes	NA	Yes	NA	NA	Yes	Fair
**Molina-Leyva and Cuenca-Barrales. Spain. 2019 [33]**	Yes	Yes	NR	Yes	Yes	NA	NA	NA	Yes	Yes	YEs	NA	NA	Yes	Fair
**Onderdijk et al. Netherlands. 2013 [34]**	Yes	Yes	NR	Yes	No	NA	NA	NA	Yes	NA	Yes	NA	NA	No	Fair
**Riis et al. Denmark. 2016 [39]**	Yes	Yes	No	Yes	No	NA	NA	NA	Yes	NA	Yes	NA	NA	No	Fair
**Sampogna et al. Italy 2019 [35]**	Yes	Yes	NR	Yes	No	NA	NA	NA	Yes	NA	Yes	NA	NA	Yes	Fair
**von der Werth et al.**	Yes	Yes	NR	Yes	No	NA	NA	NA	Yes	NA	Yes	NA	NA	No	Fair

**Denmark. 2001 [36]**															
**Vossen et al. Netherlands. 2017 [40]**	Yes	Yes	Yes	Yes	No	NA	NA	NA	Yes	NA	Yes	NA	NA	Yes	Fair

* NA, not applicable; NR, not reported
